# Optimization of H_2_O_2_ Production in Biological Systems for Design of Bio-Fenton Reactors

**DOI:** 10.3390/microorganisms12071488

**Published:** 2024-07-20

**Authors:** Peiguo Zhou, Liping Yang, Wenjing Yang, Jiaxin Hou, Ziqiao Liao

**Affiliations:** College of Ecology and Environment, Nanjing Forestry University, Nanjing 210037, China; kikicurtis_77@yeah.net (L.Y.); yyywj7089@163.com (W.Y.); houjiaxin990117@163.com (J.H.); yuki.asang.zq@hotmail.com (Z.L.)

**Keywords:** biofilms, H_2_O_2_, SBBR, Bio-Fenton, antibiotic

## Abstract

The treatment of antibiotic wastewater, which is known for its micro-toxicity, inhibition, and poor biochemistry, poses significant challenges, including complex processes, high energy demands, and secondary pollution. Bio-Fenton, a novel Fenton technology, enables the in situ production of H_2_O_2_ at near-neutral pH, having low energy requirements and sustainable properties, and reduces the hazards of H_2_O_2_ transportation and storage. We preliminary self-designed a heterogeneous Bio-Fenton reactor. An aerobic SBBR system with pure algae, pure bacteria, and bacteria–algae symbiosis was first constructed to investigate the optimal process conditions through the effects of carbon source concentration, light duration, bamboo charcoal filling rate, and dissolved oxygen (DO) content on the H_2_O_2_ production and COD removal. Second, the reactor was constructed by adding iron-carrying catalysts to remove ROX and SDZ wastewater. The results demonstrated that the optimal operating parameters of aerobic SBBR were an influent carbon source concentration of 500 mg/L, a water temperature of 20 ± 2 °C, pH = 7.5, a dissolved oxygen content of 5 mg/L, a light–dark ratio of 12 h:12 h, a light intensity of 2500 Lux, an HRT of 10 h, and a bamboo charcoal filling rate of 33%. Given these conditions, the bacterial–algal system was comprehensively found to be the most suitable biosystem for this experiment. Ultimately, the dynamically coupled Bio-Fenton process succeeded in the preliminary removal of 41.32% and 42.22% of the ROX and SDZ from wastewater, respectively.

## 1. Introduction

The pervasive detection of antibiotics in aquatic systems over recent years has emerged as a critical environmental concern. A total of 94 antibiotics were detected in water and sediment samples from seven major rivers and four sea areas in China during 2005–2016 [1], with sulfonamides (SAs), tetracyclines (TCs), macrolides (MLs), and fluoroquinolones (FQs) being the four major types of antibiotics consumed in the country [2,3]. Of these compounds, roxithromycin (ROX) and sulfadiazine (SDZ), which belong to the ML and SA classes, respectively, were found to have relatively high residual concentrations [4]. Chen et al. [5] reported the detection of MLs and TCs at concentrations of 6.05 μg·kg^−1^ and 4.95 μg·kg^−1^, respectively, in sediments of the Huangpu River. Yan et al. [6] observed the presence of SAs at concentrations up to 19.84 μg·kg^−1^ in topsoil samples collected from within the Three Gorges Reservoir area. Huang et al. [7] identified a range of pollutants, including SAs, TCs, MLs and FQs, in Guangzhou surface water. FQs were found to be present at a concentration of 645.5 ng·L^−1^, which is notable. The significant majority of discharged antibiotics enter wastewater treatment plants, yet their high toxicity and poor biochemistry hinder their entire removal using current technologies [8,9]. Furthermore, although the concentration of antibiotics in treated wastewater remains minimal, long-term discharge and accumulation contaminate surface water and groundwater and accelerate the spread of antibiotic resistance, threatening human health and safety [10,11].

Natural water biofilm is a complex biological community of living and nonliving organic and inorganic substances. Its living components are dominated by microalgae (mainly diatoms and green algae) and bacteria, and the nonliving components include inorganic minerals and metal oxides (primarily iron and manganese oxides) [12,13]. As reported previously, light has an obvious promotion effect on the degradation of organics by biofilm. However, this mechanism remains unclear: it might be related to the oxidative components (e.g., H_2_O_2_) generated by the photosynthesis by algae and others in biofilm. As biologicals such as algae produce superoxide anions (·O_2_^−^) during metabolism and·O_2_^−^ disproportionately generates H_2_O_2_ [14], natural water biofilms generate numerous reactive oxygen species (ROS) including ·O_2_^−^ and H_2_O_2_ under lighting. In addition, the ·O_2_^−^ produced by biofilms under light conditions promotes the conversion of H_2_O_2_ to ROS such as ∙OH and ^1^O_2_, which participate in the degradation of organic pollutants and the formation of Fe^2+^ [15,16]. Therefore, microscopic photo-Fenton systems may form near to or locally on biofilm surfaces in natural water bodies, promoting the degradation of pollutants.

Bio-Fenton has emerged as one of the newly developed Fenton technologies, in which microorganisms produce the Fenton reagent H_2_O_2_ through intracellular aerobic respiration using oxygen (O_2_), or Fe(II) is produced through the extracellular reduction of Fe(III) by microorganisms to drive a sustainable Fenton reaction [17]. The current methods for H_2_O_2_ production, such as electrochemical processes [18], anthraquinone autoxidation [19], and photocatalysis [20], have been found to possess inherent limitations. In addition, these chemical methods generally have disadvantages such as high cost, unfriendly environment, complex and dangerous synthesis process, and strict catalyst requirements, making them unsuitable for application in practical treatment [21,22]. However, Bio-Fenton has been proven to be an effective method for pollutant degradation and cost reduction; it harnesses the natural production of H_2_O_2_ by cells, enzymes, bacterial strains, and other biological entities during their metabolic processes [23,24]. Hua et al. [25] demonstrated that a biofilm system generated not only H_2_O_2_ but also other reactive oxygen species (ROS) under light conditions, recognizing the presence of ·OH,·O_2_^−^, and ^1^O_2_ in this system, which are directly or indirectly involved in the degradation of organic pollutants. Microorganisms, including algae, release iron and manganese ions during photosynthesis; thus, H_2_O_2_ may be converted to ·OH with strong oxidizing properties through Fenton-like reactions during the metal ion oxidation/reduction process, degrading organic pollutants [26]. Vadakke et al. [27] utilized aquatic phytoplankton to generate H_2_O_2_ with different iron particles (ferric sulfate, colloidal iron, and zeolite iron) to degrade intricate organic molecules through the production of ·OH in their presence. They proved the ability of diatoms to degrade tetracycline antibiotics and revealed that Bio-Fenton processes were the dominant removal mechanism, while adsorption and iron complex formation also contributed to the overall removal of tetracycline by diatoms. Additionally, Bio-Fenton has demonstrated effectiveness in removing challenging substances, including chloroacetanilide herbicides, azo dyes, and trichloroethylene (TCE) [28,29,30], among others.

The primary objective of this study was to establish a micro photo-Fenton system in a natural water body by leveraging biofilm and mycorrhizal algae to generate H_2_O_2_ in situ under light conditions. Concurrently, a heterogeneous Fenton reactor was developed to implement a dynamically coupled biological Fenton process for initial testing and application. A one-way variable experiment was conducted in an aerobic SBBR system in which we changed the following conditions sequentially: organic matter concentration, light exposure, bamboo charcoal filling rate, and dissolved oxygen (DO) content in a pure algae system, a pure bacterial (pure biofilm) system, and in a bacteria–algae (algae-containing biofilm) system, to determine the optimal conditions for the sustained in situ generation of H_2_O_2_ and the most suitable biological system for the subsequent experiments. Subsequently, an iron-carrying catalyst was introduced to construct a preliminary, dynamically coupled, autonomous Bio-Fenton reactor, establishing the foundation for further investigation into the effects and mechanisms of antibiotic degradation by the Bio-Fenton system.

## 2. Materials and Methods

### 2.1. Chemicals and Microorganisms

The main chemical reagents used in this experiment were glucose (C_6_H_12_O_6_), urea (H_2_NCONH_2_), K_2_Cr_2_O_7_, H_2_SO_4_, HgSO_4_, C_2_KO_4_Ti, NaNO_3_, HCl, MgSO_4_·7H_2_O, CaCl_2_·2H_2_O, citric acid (C_6_H_8_O_7_), ferric ammonium (C_6_H_8_FeNO_7_), disodium EDTA (C_10_H_14_N_2_Na_2_O_8_), Na_2_CO_3_, H_3_BO_3_, MnC_l2_·4H_2_O, ZnSO_4_·5H_2_O, Na_2_MoO_4_·5H_2_O, CuSO_4_·5H_2_O, Co (NO_3_)_2_·6H_2_O, CH_3_OH, C_2_H_3_N, C_2_H_7_NO_2_, C_2_H_4_O_2_, ROX (C_41_H_76_N_2_O_15_), and SDZ (C_10_H_10_N_4_O_2_S). The reagents above were sourced from Nanjing Chemical Reagent Co., Ltd. (Nanjing, China), Shanghai Macklin Biochemical Technology Co., Ltd. (Shanghai, China) and Sinopharm Chemical Reagent Co., Ltd. (Beijing, China). The sludge was derived from the Nanjing Chengbei Sewage Treatment Plant (Nanjing, China). *Chlorella* (green algae) was used as the primary algal source, provided by Yancheng Benro Biotechnology Co., Ltd. (Yancheng, China). The iron-carrying catalyst was purchased from Zhejiang Zhongjin Environmental Protection Technology Co., Ltd. (Ningbo, China).

### 2.2. Analytical Methods

The concentration of H_2_O_2_ was determined by spectrophotometry (T6 New Century, Beijing, China) using titanium oxalate spectrophotometry at 400 nm [31]. COD was quantified by spectrophotometry (T6 New Century, Beijing, China) at 440 nm, employing the fast digestion spectrophotometric method (Chinese EPA 2002). Total nitrogen (TN) was determined via UV spectrophotometry (T6 New Century, Beijing, China) using the alkaline potassium persulfate digestion method at 220 nm and 275 nm. An LC-5060 UV/Vis detector (Tengzhou, China) was used to analyze the concentrations of ROX and SDZ at 210 nm and 270 nm, respectively [32]. The microorganisms present in suspension sludge were observed under a light microscope (XSP-8CA, Fuzhou, China). High-resolution micromorphological images of bamboo charcoal surface were obtained using a cold field-emission scanning electron microscope (SEM, Regulus 8100, Tokyo, Japan) before and following the coating process.

### 2.3. The Characterization of Wastewater

During the preconstruction stage of the Bio-Fenton system, the simulated domestic sewage for the experiments was prepared by diluting glucose, ammonium chloride, and dipotassium hydrogen phosphate with tap water after being left to stand for 1–2 days to reduce the stimulation of microorganisms by residual chlorine and control carbon, nitrogen, and phosphorus in a ratio of 100:5:1. After system construction, the simulated antibiotic wastewater used was formulated by diluting ROX and SDZ with static tap water. The concentration range of ROX and SDZ in the formulated wastewater was 2.5 mg/L to 10 mg/L, which was based on the physical treatment of ROX and SDZ wastewater before it underwent biotreatment.

### 2.4. Dynamically Coupled Bio-Fenton Reactor

**Figure 1:**
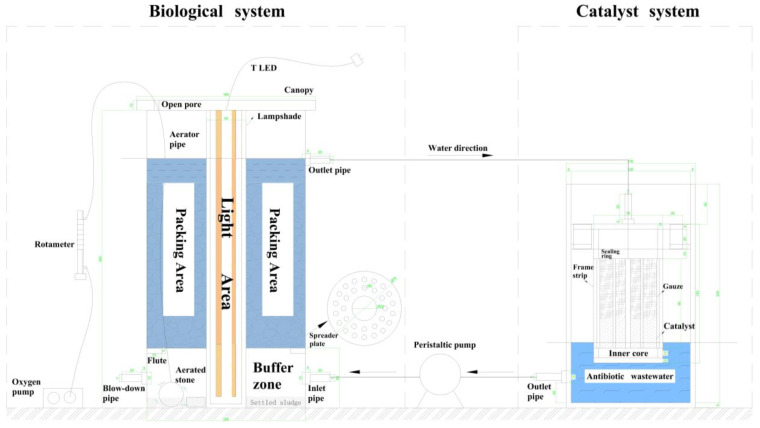
Schematic diagram of the dynamically coupled Bio-Fenton reactor.

As illustrated in Figure 1, the device was constructed from acrylic material and comprised two distinct sections: a biological system and a catalyst system. The biological system section was constructed upon an SBBR reactor, which primarily consisted of an influent buffer, packing area, and light area, with components that could be flexibly dismantled in each section. The total effective volume of the system was 4.71 L, of which 3.58 L was the effective filling volume. The bamboo charcoal had a particle size of 1 cm to 3 cm, a fill height of 12 cm, a fill volume of 1.20 L (obtained by the draining volume method), a total mass of 1.21 kg, and a porosity of 53.05%. The catalyst system consisted of an external slotted column and an internal removable hollow core.

In this device, the influent buffer zone mitigated the adverse effects of wastewater on the biological system. The buffer zone comprised a spherical aeration stone, with a diameter of 4 cm, and a water cloth plate, with fine holes set 6 cm high. These components facilitated the water flow and aeration dispersion, ensuring an optimal distribution throughout the buffer zone. The light source comprised 3 cm external lampshades and two internal 6 W LED plant growth lamps, emitting light at approximately 460 nm. The light intensity could be adjusted. The biofilm reactor was designed with an upward flow, a downward inlet, and an upward outlet. Once the overflow port became full, water was automatically directed into the inner core, loaded with catalysts. The right side of the device accumulated water, and a peristaltic pump pumped the water at a uniform rate into the biofilm reactor through a controlled flow rate. This process was designed to circulate water, continuously degrade pollutants, and realize the dynamic coupling of the Bio-Fenton process.

Algal amplification, biofilm hanging, and other preparations were carried out during the reactor start-up period. By measuring the effluent COD concentration daily and observing the growth of sludge and packing microorganisms under optical microscopy and SEM, we evaluated whether the reactor start-up had succeeded, and the total running time was 25 days. After success, research on factors affecting H_2_O_2_ production was carried out first in the biosystem reactor. Afterward, preliminary experiments were conducted on the degradation of antibiotic wastewater in dynamically coupled the Bio-Fenton reactor.

## 3. Results and Discussion

### 3.1. Characterization of Fillers and Microorganisms

**Figure 2:**
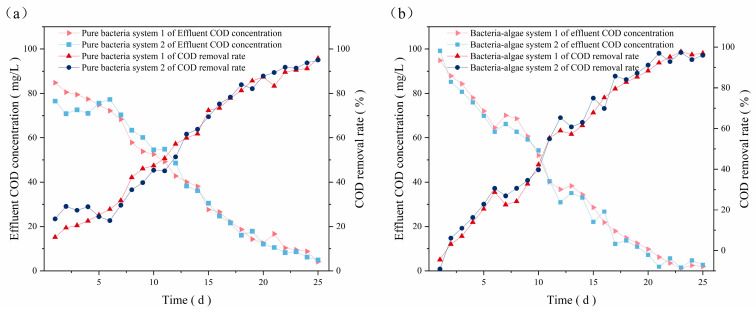
Changes in effluent COD concentration and removal rate of (**a**) bacterial system and (**b**) bacteria–algae system during membrane hanging.

Membrane hanging was conducted in four SBBR reactors, designated as pure bacterial system 1, pure bacterial system 2, and bacteria–algae system 1, and bacteria–algae system 2, respectively. Figure 2 demonstrates the variation in the degradation capacity during the preliminary membrane hanging of the SBBR reactor with glucose-simulated wastewater. After 20 days, the COD removal rate of the bacterial–algal system was 92.74%, while that of the pure bacterial system reached 90% at 23 days. By the late stage of film hanging, the COD removal rate of the bacterial–algal system was maintained above 96%, and the pure bacterial system remained in the fluctuation stage, with a rate of 90%. This indicated that the symbiotic bacterial–algal system degraded and removed pollutants more efficiently. Consequently, it was clear that the SBBR reactors had a certain level of degradation capacity, and the effluent indicators met the expected demands.

**Figure 3:**
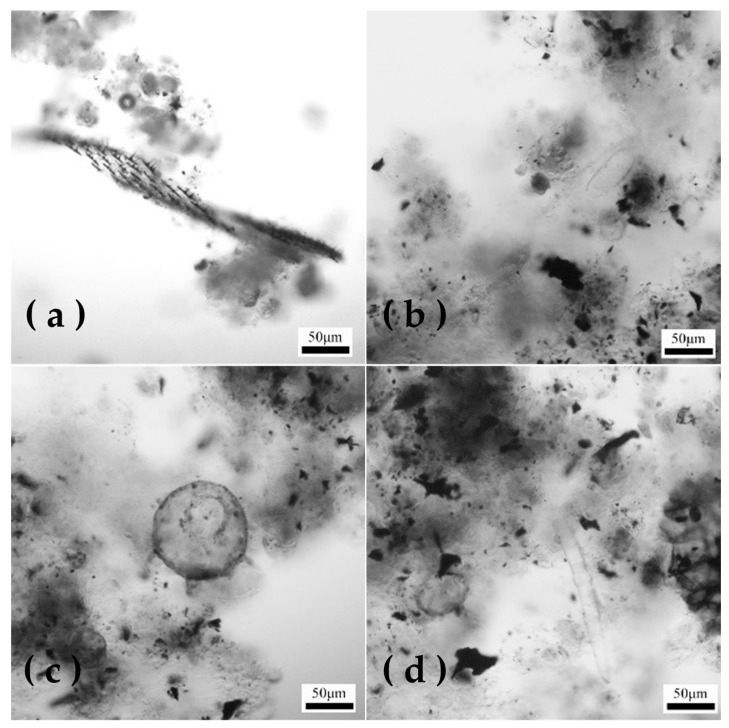
Microbiological microscopy images of inoculated sludge at (**a**) initial stage; (**b**) prophase; (**c**) middle stage; (**d**) late stage.

The growth of microorganisms at different stages after sludge inoculation is shown in Figure 3, which reflects the reactor degradation capacity and water quality characteristics. In Figure 3a, *Flagellate* microorganisms can be observed, with no obvious sparse material; meanwhile, the microbial flora in sludge was single-species in nature, and the water quality purification was poor. Figure 3b shows the pre-inoculation stage of sludge, which hosted microorganisms like *bean-shaped worms*, *lacewings*, and *nematodes*, showing that the sludge flocs were finely flaked and that the active sludge and water quality conditions were poor this time. Considering the COD effluent concentration, it could be concluded that the reactor was still in the initial stage, with a COD removal rate of sewage of less than 10% and showing weak degradation ability [33]. Figure 3c demonstrates the middle stage of sludge inoculation, where carnivorous protozoa such as *sunworms* were observed, and COD removal reached 50% this time, meaning that water quality tended to improve with increased treatment capacity of the system [34]. Figure 3d demonstrates the presence of a small number of organisms similar to *Epistylis* and *bellworms* in the later stages of sludge inoculation. Generally, when these two types of protozoa were present, the activated sludge performed well, the water had a low BOD [35], and the COD removal rate remained stable at more than 90% in the biological treatment system. Additionally, the sludge floc was obvious to the naked eye, showing good settling performance and a compact bacterial colloid structure. The sludge was mixed with bamboo charcoal powder to a grey-brown color. It was found that within three weeks of system operation, the activated sludge properties had become stable, showing adaptability to the influent conditions. In addition to the degradation of COD, the discharge water quality met the standard, and the reactor started successfully.

**Figure 4:**
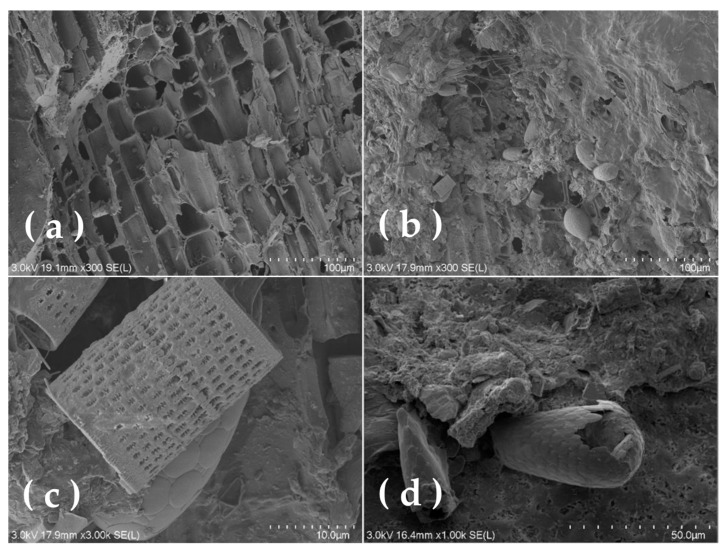
Microbial attachment on the bamboo charcoal surface (**a**) before and (**b**) after film hanging; (**c**) hollow cylindric microorganisms and (**d**) spotted conical microorganisms.

Figure 4 demonstrates the microbial adhesion to the surface of the bamboo charcoal before and after filming. Figure 4a,b clearly show that the internal pores of the clean bamboo charcoal without filming were in the shape of hollow bamboo joints, with no signs of microbial adhesion around them. In the middle and late stages, bamboo charcoal pores were gradually filled with mainly filamentous microorganisms, and the interpore spaces were covered with an adhered biofilm. Figure 4c,d show roller-shaped and oval-shaped objects with mottled patterns, which we suspected were microorganisms. Comparing the film-carrying condition of the bamboo charcoal pores before and after coating, along with the yellowish-brown film visible on the surface of bamboo charcoal and the slippery feel, it was concluded that the biofilm on the filler of system was mature, and the coating of the bamboo charcoal was successful.

### 3.2. Production of H_2_O_2_ by Biological Systems

#### 3.2.1. Pure Algae System

**Figure 5:**
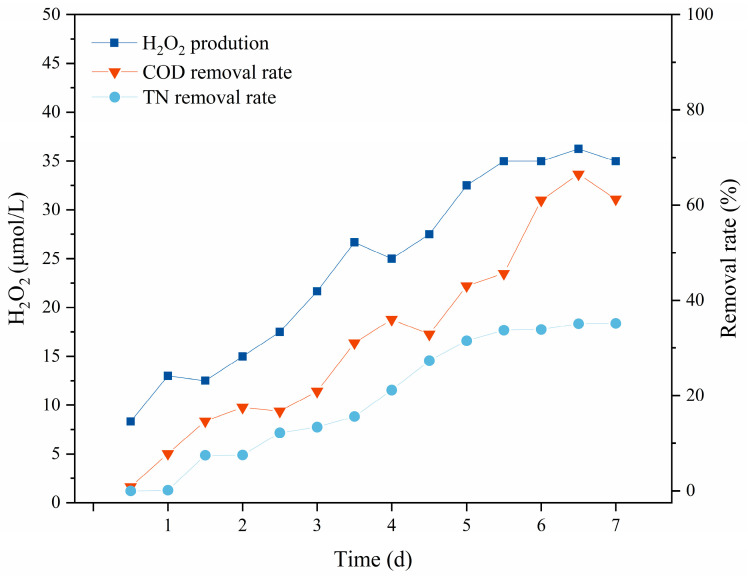
Changes in hydrogen peroxide production and COD and TN removal rates in pure algae system.

The H_2_O_2_ generation, COD removal, and TN removal in the pure algae system were measured over 7 days, and the data are shown in Figure 5. H_2_O_2_ production showed an increasing trend as incubation progressed with algae, which gradually rose from 5 μmol/L to 35 μmol/L. Indeed, under photosynthetic conditions, algal cells proliferated in the logarithmic phase and constantly produced ROS molecules such as ·O_2_^−^ and H_2_O_2_. Then, the concentration of hydrogen peroxide in the system stabilized, which may have been related to the algae’s antioxidant protection mechanism. When algae’s ability to generate reactive oxygen molecules is inhibited, metabolic disorders or cellular deformation occur in the algal cells. However, an excess of strong extracellular oxidative substances further inhibits cellular activity. At this time, the algae initiate a free radical scavenging mechanism to reduce oxidative stress through the decomposition of excess H_2_O_2_ [36,37]. In general, the rate of H_2_O_2_ production in the pure algae system first increased and subsequently decreased throughout the incubation period. COD removal was as high as 66.48%, whereas TN removal was only as high 35.17%, due to the nitrogen limitation that is induced when the N/P ratio is less than 5:1 [38]. By the fifth day, the rate of H_2_O_2_ production in the system and the removal efficiency of TN and COD by the algae reached a plateau, indicating a stagnant or even declining trend. This was attributed to the fact that some algal cells had begun to enter the decline phase from the stable phase. Additionally, the single algal system lacked continuous access to CO_2_, so they could not degrade most pollutants cyclically.

#### 3.2.2. Pure Bacterial System

**Figure 6:**
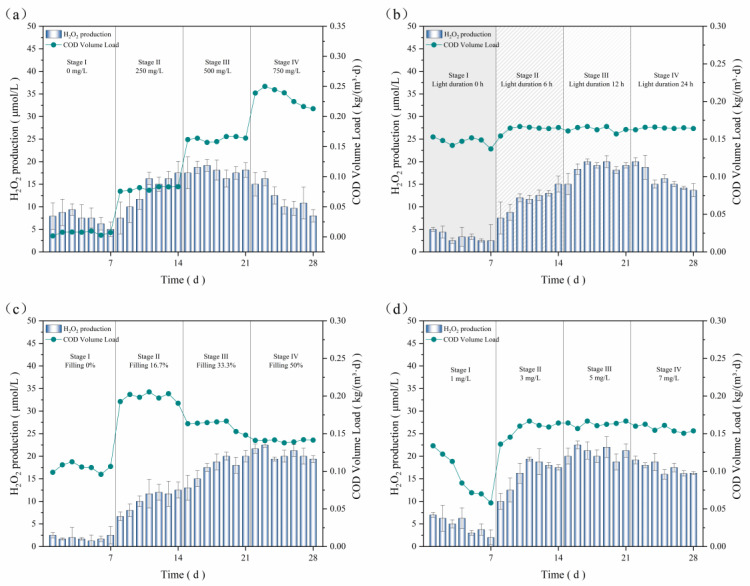
Influence of (**a**) carbon source concentration; (**b**) light duration; (**c**) bamboo charcoal filling rate; (**d**) dissolved oxygen on H_2_O_2_ production and COD volume loading.

The optimal influent carbon source concentration in the pure bacterial system was investigated at a fixed water temperature of 20 °C, a pH = 7.5, a DO of 3 mg/L, a 12 h:12 h light–dark ratio, a light intensity of 2500 Lux, and a bamboo charcoal filling rate of 33%, with results shown in Figure 6a. The figure reveals that the highest H_2_O_2_ production (19.17 μmol/L in stage III) was achieved with an influent carbon source concentration of 500 mg/L, while the effluent COD concentration was maintained below 15 mg/L. The average H_2_O_2_ production and COD volumetric loading in the pure bacterial system gradually increased with the increase in glucose concentration in stages I to III, while the H_2_O_2_ production of the system gradually decreased without an added carbon source, and the lowest production was only 5 μmol/L. This indicated that the uptake of nutrients was essential for the growth metabolism in the bacteria–algae systems, ensuring favorable microorganism activity for photosynthesis and ROS production. Upon entering stage IV, the average H_2_O_2_ production and COD volumetric loading exhibited a declining trend, with the lowest values observed at 8 μmol/L and 0.21 kg/(m^3^·d), respectively. This was due to the influent carbon increasing at this stage, which resulted in an organic load of up to 0.25 kg/(m^3^·d). The high organic load inhibited the growth of microorganisms, which in turn led to a decrease in their abilities to degrade COD and produce H_2_O_2_. Consequently, an influent carbon source of 500 mg/L was selected for subsequent experiments, as an insufficient or excessive load was detrimental to the microbial retention of viability.

According to Figure 6b, the average H_2_O_2_ production rose significantly as the light duration increased from 0 h to 12 h, where we observed that H_2_O_2_ production increased from 6.83 μmol/L (stage I) to a peak of 21.49 μmol/L (stage III), with no further increases observed beyond this duration. This phenomenon can be interpreted as the prolonged light inhibiting microbial respiration and decomposing the generated H_2_O_2_, but, compared with the dark environment, continuous light conditions were more conducive to the conversion of free radicals and the photolysis of a part of the organic matter in the system. Hence, the decrease in H_2_O_2_ production did not impact the COD removal efficiency. The optimal light duration obtained from this experiment is 12 h, corresponding to a light–dark ratio of 12 h:12 h.

The influence of the bamboo charcoal filling rate on the average H_2_O_2_ production and COD volumetric loading was investigated, and the resulting data are presented in Figure 6c. It shows that as the bamboo charcoal filling rate rose, the system’s average production of H_2_O_2_ gradually increased, while the COD volumetric loading gradually decreased. As the bamboo charcoal filling rate increased from 0% to 50%, the average H_2_O_2_ production experienced a significant increase, rising from 1.89 μmol/L to 20.60 μmol/L. Nevertheless, the COD volumetric loading exhibited a slight decline when the filling rate reached 50%, which was primarily attributable to the filling coefficient being directly proportional to the effective volume of the reactor. In stage IV of the system, the bamboo charcoal filling coefficient was the highest (50%), resulting in the largest number of biofilms and almost 100% organics’ removal, leading to a lower COD volumetric loading.

Conversely, excessive filler was not conducive to drainage or mixing within the system. Furthermore, there was no significant difference in the H_2_O_2_ production between the filler rates of 33.33% and 50%, while the degradation of organics stabilized at more than 96%. Considering financial costs and operational efficacy, the optimal bamboo charcoal filling rate was determined to be 33.33%, equaling a volume of bamboo charcoal of 1 L in this system.

Figure 6d shows the effect of the DO on the average H_2_O_2_ production and COD volumetric loading in the pure bacteria system. A marked increase in the average H_2_O_2_ production was observed as DO levels rose from 1 mg/L to 5 mg/L, which subsequently declined at elevated DO levels. At a dissolved oxygen level of 1 mg/L, both the H_2_O_2_ production and COD volumetric loading of the system gradually declined over the day, down to 2 μmol/L and 0.058 kg/(m^3^·d). This phenomenon could be attributed to the inadequate DO, which impeded the metabolic activity of aerobic microorganisms. The aerobic system, in general, necessitates the DO to be maintained at 3 mg/L or above. Entering stage II, the pure bacteria system first adapted to gradually recover from the low oxygen level, and then H_2_O_2_ production and COD volumetric loading stabilized at 18 μmol/L and 0.164 kg/(m^3^·d), respectively. Continuously increasing the DO level in the system resulted in an increase in H_2_O_2_ production by 2 μmol/L on average, while COD removal was maintained at over 98%. The average H_2_O_2_ production and COD volumetric loading decreased when the DO was raised to 7 mg/L in the system, which was attributed to the elevated dissolved oxygen levels disrupting the stability of the sludge flocs, leading to the oxidation of the sludge and biofilm, thereby highlighting the pivotal role of DO in influencing the degradation of organic matter by biofilm. The optimal DO level of 5 mg/L was used in the subsequent experiments.

#### 3.2.3. Bacteria–Algae System

**Figure 7:**
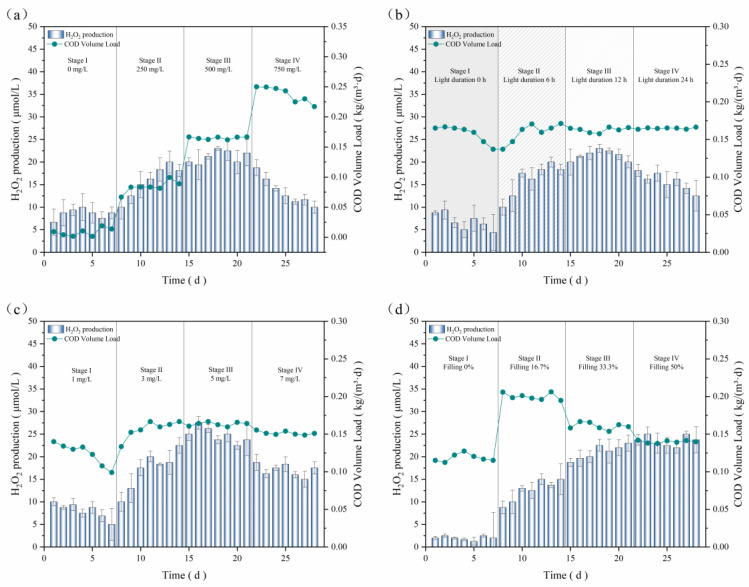
Influence of (**a**) carbon source concentration; (**b**) light duration; (**c**) bamboo charcoal filling rate; (**d**) dissolved oxygen on H_2_O_2_ production and COD volume loadings.

Considering the initial reaction conditions of the pure bacterial system as a reference, the effects of altering the carbon source on H_2_O_2_ production and COD volumetric loading of the bacterial–algal system were examined, and the obtained data are organized and presented in Figure 7a. From stages I to III, the average H_2_O_2_ production and COD volumetric loading in the bacteria–algae system gradually increased with the increase in glucose concentration from 8.55 μmol/L to 21.16 μmol/L and from 0.008 kg/(m^3^·d) to 0.165 kg/(m^3^·d), respectively. A comparison with the data in Section 3.2.2 shows that both the average H_2_O_2_ production and COD volumetric loading in the bacteria–algae system were higher than those of the pure bacterial system. This was due to the mutualistic symbiosis between the bacteria and algae, which facilitated energy replenishment and transformation. Thee metabolism and capacity of the bacteria–algae system to treat wastewater with the same concentration of carbon were more significant. With a further increase in the carbon source concentration to 750 mg/L, both H_2_O_2_ production and COD volumetric loading began to decrease, to 10 μmol/L and 0.217 kg/(m^3^·d), respectively, yet remained higher than those in the pure bacterial system by 1.75 μmol/L and 0.005 kg/(m^3^·d), indicating that the bacteria–algae system had greater stability at higher organic loadings. Therefore, 500 mg/L was selected as the optimum carbon source concentration.

The effects of the light duration and bamboo charcoal filling rate on the bacteria–algae system were the same as those in the pure bacterial system. As shown in Figure 7b, it can be seen that the average H_2_O_2_ production increased gradually when the light duration was increased from 0 h to 12 h, beyond which it decreased. The average H_2_O_2_ production and COD volumetric loading of the system were optimal when the light duration was 12 h (stage III), which stabilized at 21.49 μmol/L and 0.163 kg/(m^3^·d), respectively. At stage IV, the average H_2_O_2_ production gradually reduced to 15.68 μmol/L, which decreased at a faster rate than that in the pure bacterial system, since the continuous light disrupted the alternating cycle of photosynthesis and respiration, which mainly affected the physiological cycle of the algal cells in the bacteria–algae system [39]. Consequently, in the subsequent experiments, we used an optimal light duration of 12 h. The effect of the bamboo charcoal filling rate on the H_2_O_2_ production and COD volumetric loading of the bacterial-algae system is presented in Figure 7c, and 33.33% was selected as the optimal rate. Under this condition, the average H_2_O_2_ production was 21.02 μmol/L in this system, which was 3 μmol/L higher than that in the pure bacterial system.

In Figure 7d, it can observed that the average H_2_O_2_ production increased up to 24.82 μmol/L when the DO content elevated from 1 mg/L to 5 mg/L but decreased at higher DO contents. Combined with the findings in the existing literature, a high DO content could inhibit algal photosynthesis [40] or lead to the blowing off of carbon dioxide [41], resulting in a significant loss of inorganic carbon and restricted algal growth due to the lack of available inorganic carbon. In summary, the natural water biofilm system under light exposure probably generated several reactive substances that were affected by DO, which then facilitated the oxidative decomposition of the pollutants. Nevertheless, hyperintense aeration may collapse the algae system, so we chose an optimum DO level of 5 mg/L.

### 3.3. Dynamically Coupled Bio-Fenton Process

**Figure 8:**
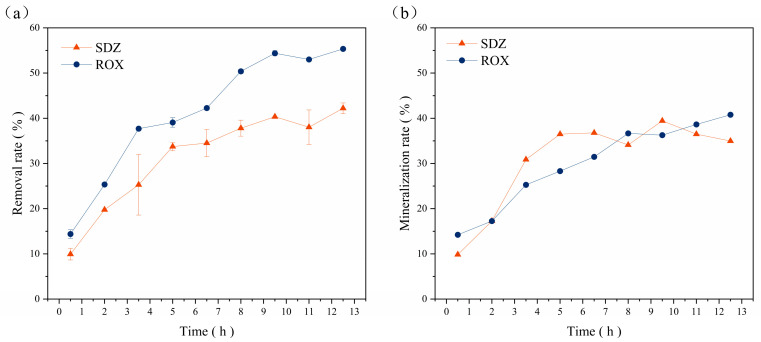
(**a**) Removal rate and (**b**) mineralization rate of SDZ and ROX: the influent ROX and SDZ concentrations were 10 mg/L, glucose addition was 0.5 g/L, HRT was 4.5 h, water temperature was 20 ± 2 °C, pH = 7.5, DO was 5 mg/L, light intensity was 1000 Lux, and 50 g of iron-carrying catalyst was used.

Separate reactors were used for the degradation of ROX and SDZ wastewater (total of two reactors). Each reactor was continuously supplied with dynamic water, influent ROX and SDZ concentrations of 10 mg/L, glucose addition of 0.5 g/L, an HRT of 4.5 h, a water temperature of 20 ± 2 °C, a pH = 7.5, a DO of 5 mg/L, a light intensity of 1000 Lux, and 50 g of iron-carrying catalyst. After 0.5 h of influent supply, samples were taken in 1.5 h intervals to determine the concentrations of ROX, SDZ, and TOC. The experiment was conducted for 12 h per day over 5 days. As shown in Figure 8, the average removal rates of ROX and SDZ by this system were 55.34% and 42.22%, respectively, and the average mineralization rates were 40.78% and 34.97%.

## 4. Conclusions

The H_2_O_2_ production patterns of different biological systems were explored in an aerobic SBBR system; subsequently, a dynamically coupled Bio-Fenton reactor was constructed by adding iron-carrying catalysts. We constructed three different biological systems of pure algae, pure bacteria, and bacteria–algae, and we characterized the microbial phases via optical microscopy and SEM. The results confirmed the sludge inoculation status and showed that microbes were tightly attached to the surface of the bamboo charcoal, indicating the SBBR start-up was a success. Subsequently, the optimal conditions for the sustained in situ generation of H_2_O_2_ in both the pure bacterial system and bacterial–algal system were determined using a one-way variability method as a glucose concentration of 500 mg/L, a water temperature of 20 ± 2 °C, a pH = 7.5, a DO of 5 mg/L, a light–dark ratio of 12h:12h, a light intensity of 2500 Lux, an HRT of 10 h, and a bamboo charcoal filling rate of 33%. A comparison of these three biological systems revealed that the H_2_O_2_ production was as follows: pure algae system > bacteria–algae system > pure bacterial system; COD removal, highest to lowest, was bacteria–algae system > pure bacterial system > pure algal system. Thus, the bacterial–algal system was selected as the most suitable biological system for our subsequent experiments. Furthermore, a coupled Bio-Fenton reactor was constructed by adding iron-carrying catalysts to the optimum system, which was followed by preliminary degradation experiments on ROX and SDZ wastewater, which obtained average removal rates of 41.32% and 42.22% and average mineralization rates of 40.78% and 34.97% for ROX and SDZ, respectively. These outcomes demonstrate that the designed dynamically coupled Bio-Fenton reactor was a success and shows value for practical applications.

## Data Availability

The original contributions presented in the study are included in the article, further inquiries can be directed to the corresponding author.

## References

[1] Li Z., Li M., Zhang Z., Li P., Zang Y., Liu X. (2020). Antibiotics in Aquatic Environments of China: A Review and Meta-Analysis. Ecotoxicol. Environ. Saf..

[2] Zhang Q.-Q., Ying G.-G., Pan C.-G., Liu Y.-S., Zhao J.-L. (2015). Comprehensive Evaluation of Antibiotics Emission and Fate in the River Basins of China: Source Analysis, Multimedia Modeling, and Linkage to Bacterial Resistance. Environ. Sci. Technol..

[3] Gao L., Shi Y., Li W., Niu H., Liu J., Cai Y. (2012). Occurrence of Antibiotics in Eight Sewage Treatment Plants in Beijing, China. Chemosphere.

[4] Oberoi A.S., Jia Y., Zhang H., Khanal S.K., Lu H. (2019). Insights into the Fate and Removal of Antibiotics in Engineered Biological Treatment Systems: A Critical Review. Environ. Sci. Technol..

[5] Chen K., Zhou J.L. (2014). Occurrence and Behavior of Antibiotics in Water and Sediments from the Huangpu River, Shanghai, China. Chemosphere.

[6] Yan M., Xu C., Huang Y., Nie H., Wang J. (2018). Tetracyclines, Sulfonamides and Quinolones and Their Corresponding Resistance Genes in the Three Gorges Reservoir, China. Sci. Total Environ..

[7] Huang Y.-H., Liu Y., Du P.-P., Zeng L.-J., Mo C.-H., Li Y.-W., Lü H., Cai Q.-Y. (2019). Occurrence and Distribution of Antibiotics and Antibiotic Resistant Genes in Water and Sediments of Urban Rivers with Black-Odor Water in Guangzhou, South China. Sci. Total Environ..

[8] Phoon B.L., Ong C.C., Mohamed Saheed M.S., Show P.-L., Chang J.-S., Ling T.C., Lam S.S., Juan J.C. (2020). Conventional and Emerging Technologies for Removal of Antibiotics from Wastewater. J. Hazard. Mater..

[9] Xu J., Zhang Y., Zhou C., Guo C., Wang D., Du P., Luo Y., Wan J., Meng W. (2014). Distribution, Sources and Composition of Antibiotics in Sediment, Overlying Water and Pore Water from Taihu Lake, China. Sci. Total Environ..

[10] Li J., Qiu X., Ren S., Liu H., Zhao S., Tong Z., Wang Y. (2023). High Performance Electroactive Ultrafiltration Membrane for Antibiotic Resistance Removal from Wastewater Effluent. J. Membr. Sci..

[11] Rambabu K., Banat F., Pham Q.M., Ho S.-H., Ren N.-Q., Show P.L. (2020). Biological Remediation of Acid Mine Drainage: Review of Past Trends and Current Outlook. Environ. Sci. Ecotechnol..

[12] Beck A.J., Janssen F., Polerecky L., Herlory O., De Beer D. (2009). Phototrophic Biofilm Activity and Dynamics of Diurnal Cd Cycling in a Freshwater Stream. Environ. Sci. Technol..

[13] Wimpenny J. (1996). Ecological Determinants of Biofilm Formation. Biofouling.

[14] Clark C.D., De Bruyn W.J., Hirsch C.M., Jakubowski S.D. (2010). Hydrogen Peroxide Measurements in Recreational Marine Bathing Waters in Southern California, USA. Water Res..

[15] Fujii M., Dang T.C., Rose A.L., Omura T., Waite T.D. (2011). Effect of Light on Iron Uptake by the Freshwater Cyanobacterium *Microcystis aruginosa*. Environ. Sci. Technol..

[16] Reczek C.R., Chandel N.S. (2015). ROS-Dependent Signal Transduction. Curr. Opin. Cell Biol..

[17] Kahoush M., Behary N., Cayla A., Nierstrasz V. (2018). Bio-Fenton and Bio-Electro-Fenton as Sustainable Methods for Degrading Organic Pollutants in Wastewater. Process Biochem..

[18] Santos G.O.S., Cordeiro-Junior P.J.M., Sánchez-Montes I., Souto R.S., Kronka M.S., Lanza M.R.V. (2022). Recent Advances in H_2_O_2_ Electrosynthesis Based on the Application of Gas Diffusion Electrodes: Challenges and Opportunities. Curr. Opin. Electrochem..

[19] Kurc L., Páter M., Červený L. (2003). Activity of Basic Catalysts in Oxidation of 2-Ethyl-5,6,7,8-Tetrahydro-9,10-Anthrahydroquinone. J. Mol. Catal. A Chem..

[20] Hu S., Sun X., Zhao Y., Li W., Wang H., Wu G. (2020). The Effective Photocatalytic Water Splitting to Simultaneously Produce H_2_ and H_2_O_2_ over Pt Loaded K-g-C_3_N_4_ Catalyst. J. Taiwan Inst. Chem. Eng..

[21] Shi K., Wang J., Yin L., Xu Y., Kong D., Li H., Zhang Y., He H., Yang S., Ni L. (2023). Photocatalysis Combined with Microalgae to Promote the Degradation and Detoxification of Tetracycline Hydrochloride. Bull. Environ. Contam. Toxicol..

[22] Kim D., Watanabe M., Nakayasu Y., Kohata K. (2005). Changes in O_2_- and H_2_O_2_ Production by Chattonella Antiqua during Diel Vertical Migration under Nutrient Stratification. Aquat. Microb. Ecol..

[23] Ghatge S., Yang Y., Ko Y., Yoon Y., Ahn J.-H., Kim J.J., Hur H.-G. (2022). Degradation of Sulfonated Polyethylene by a Bio-Photo-Fenton Approach Using Glucose Oxidase Immobilized on Titanium Dioxide. J. Hazard. Mater..

[24] Feng M., Xie Y., Mao W., Lu Y., Wang Y., Li H., Zhang C. (2023). Efficient Biodegradation of Tris-(2-Chloroisopropyl) Phosphate by a Novel Strain Amycolatopsis Sp. FT-1: Process Optimization, Mechanism Studies and Toxicity Changes. J. Hazard. Mater..

[25] Hua X., Li M., Su Y., Dong D., Guo Z., Liang D. (2012). The Degradation of Linear Alkylbenzene Sulfonate (LAS) in the Presence of Light and Natural Biofilms: The Important Role of Photosynthesis. J. Hazard. Mater..

[26] Peng Y.-Y., Gao F., Yang H.-L., Wu H.-W.-J., Li C., Lu M.-M., Yang Z.-Y. (2020). Simultaneous Removal of Nutrient and Sulfonamides from Marine Aquaculture Wastewater by Concentrated and Attached Cultivation of Chlorella Vulgaris in an Algal Biofilm Membrane Photobioreactor (BF-MPBR). Sci. Total Environ..

[27] Vadakke Pariyarath R., Inagaki Y., Sakakibara Y. (2021). Phycoremediation of Tetracycline via Bio-Fenton Process Using Diatoms. J. Water Process Eng..

[28] Yang Y., Ghatge S., Ko Y., Yoon Y., Ahn J.-H., Kim J.J., Hur H.-G. (2022). Non-Specific Degradation of Chloroacetanilide Herbicides by Glucose Oxidase Supported Bio-Fenton Reaction. Chemosphere.

[29] Eskandarian M., Mahdizadeh F., Ghalamchi L., Naghavi S. (2014). Bio-Fenton Process for Acid Blue 113 Textile Azo Dye Decolorization: Characteristics and Neural Network Modeling. Desalin. Water Treat..

[30] Ravi S., Lonappan L., Touahar I., Fonteneau É., Vaidyanathan V.K., Cabana H. (2020). Evaluation of Bio-Fenton Oxidation Approach for the Remediation of Trichloroethylene from Aqueous Solutions. J. Environ. Manag..

[31] Lukes P., Appleton A.T., Locke B.R. (2004). Hydrogen Peroxide and Ozone Formation in Hybrid Gas–Liquid Electrical Discharge Reactors. IEEE Trans. Ind. Applicat..

[32] Li W., Lyu B., Li J., Korshin G.V., Zhang M., Zhang Y., Li P., Han J. (2020). Phototransformation of Roxithromycin in the Presence of Dissolved Organic Matter: Characteriazation of the Degradation Products and Toxicity Evaluation. Sci. Total Environ..

[33] Papadimitriou C., Palaska G., Lazaridou M., Samaras P., Sakellaropoulos G.P. (2007). The Effects of Toxic Substances on the Activated Sludge Microfauna. Desalination.

[34] Curds C.R. (1982). The Ecology and Role of Protozoa in Aerobic Sewage Treatment Processes. Annu. Rev. Microbiol..

[35] Chen S., Xu M., Cao H., Zhu J., Zhou K., Xu J., Yang X., Gan Y., Liu W., Zhai J. (2004). The Activated-Sludge Fauna and Performance of Five Sewage Treatment Plants in Beijing, China. Eur. J. Protistol..

[36] Lu I.-F., Sung M.-S., Lee T.-M. (2006). Salinity Stress and Hydrogen Peroxide Regulation of Antioxidant Defense System in Ulva Fasciata. Mar. Biol..

[37] Zandalinas S.I., Mittler R. (2018). ROS-Induced ROS Release in Plant and Animal Cells. Free Radic. Biol. Med..

[38] Rasdi N.W., Qin J.G. (2015). Effect of N:P Ratio on Growth and Chemical Composition of Nannochloropsis Oculata and Tisochrysis Lutea. J. Appl. Phycol..

[39] Ralph P.J., Gademann R. (2005). Rapid Light Curves: A Powerful Tool to Assess Photosynthetic Activity. Aquat. Bot..

[40] Kesaano M., Sims R.C. (2014). Algal Biofilm Based Technology for Wastewater Treatment. Algal Res..

[41] Zhang H., Gong W., Bai L., Chen R., Zeng W., Yan Z., Li G., Liang H. (2020). Aeration-Induced CO_2_ Stripping, Instead of High Dissolved Oxygen, Have a Negative Impact on Algae–Bacteria Symbiosis (ABS) System Stability and Wastewater Treatment Efficiency. Chem. Eng. J..

